# Clofarabine versus fludarabine‐based reduced‐intensity conditioning regimen prior to allogeneic transplantation in adults with AML/MDS

**DOI:** 10.1002/cam4.880

**Published:** 2016-10-17

**Authors:** Patrice Chevallier, Myriam Labopin, Regis Peffault de La Tour, Bruno Lioure, Claude‐Eric Bulabois, Anne Huynh, Didier Blaise, Pascal Turlure, Etienne Daguindau, Natacha Maillard, Ibrahim Yakoub‐Agha, Gaelle Guillerm, Jeremy Delage, Nathalie Contentin, Jacques‐Olivier Bay, Florence Beckerich, Jean‐Henri Bourhis, Marie Detrait, Stéphane Vigouroux, Sylvie François, Faezeh Legrand, Thierry Guillaume, Mohamad Mohty, the SFGM‐TC

**Affiliations:** ^1^ Hematology DepartmentCHU Hotel‐DieuNantesFrance; ^2^ Université Pierre & Marie CurieParisFrance; ^3^ INSERM, UMRs 938ParisFrance; ^4^ Hôpital Saint‐Antoine, AP‐HPParisFrance; ^5^ Hematology DepartmentHôpital Saint‐LouisParisFrance; ^6^ Hematology DepartmentCHRU HautepierreStrasbourgFrance; ^7^ Hematology DepartmentCHUGrenobleFrance; ^8^ Hematology DepartmentCentre Anti‐cancéreuxToulouseFrance; ^9^ Hematology DepartmentInstitut Paoli‐CalmetteMarseilleFrance; ^10^ Hematology DepartmentCHULimogesFrance; ^11^ Hematology DepartmentCHUBesançonFrance; ^12^ Hematology DepartmentCHUPoitiersFrance; ^13^ CHU de Lille, LIRIC INSERM U995Université Lille2LilleFrance; ^14^ Hematology DepartmentCHUBrestFrance; ^15^ Hematology DepartmentCHUMontpellierFrance; ^16^ Hematology DepartmentCHURouenFrance; ^17^ Hematology DepartmentCHUClermont‐FerrandFrance; ^18^ Hematology DepartmentCHU Henri MondorCreteilFrance; ^19^ Hematology DepartmentIGRVillejuifFrance; ^20^ Hematology DepartmentCHULyonFrance; ^21^ Hematology DepartmentCHUBordeauxFrance; ^22^ Hematology DepartmentCHUAngersFrance; ^23^ Hematology DepartmentCHUNiceFrance

**Keywords:** Acute myeloid leukemia, allogeneic stem cell transplantation, clofarabine, fludarabine, myelodysplastic syndrome, reduced‐toxicity conditioning regimen

## Abstract

We have retrospectively compared survivals between acute myeloid leukemia (AML)/myelodysplastic syndrome (MDS) patients who received either a clofarabine/busulfan (CloB2A2) or a fludarabine/busulfan (FB2A2) RIC regimen for allogeneic stem cell transplantation. Between 2009 and 2014, 355 allotransplanted cases were identified from the SFGM‐TC registry as having received either the FB2A2 (*n* = 316, 56% males, median age: 59.2 years, AML 78.5%, first complete remission [CR1] 72%, median follow‐up: 20 months) or the CloB2A2 (*n* = 39, 62% males, median age: 60.8 years, AML 62%, CR1 69%, median follow‐up: 22.4 months) RIC regimen. In multivariate analysis, FB2A2 was associated with significant lower overall survival (OS, HR: 2.14; 95%CI: 1.05–4.35, *P* = 0.04) and higher relapse incidence (RI, HR: 2.17; 95%CI: 1.02–4.61, *P* = 0.04) and a trend for lower leukemia‐free survival (LFS, HR: 1.75; 95%CI: 0.94–3.26, *P* = 0.08). These results were confirmed using a propensity score‐matching strategy. However, when considering AML and MDS patients separately, the benefit of the CLOB2A2 regimen was restricted to AML patients (2‐year OS FB2A2: 38% [14.5–61.6] vs. CloB2A2: 79.2% [62.9–95.4], *P* = 0.01; 2‐year LFS FB2A2: 38% [16–59.9] vs. CloB2A2: 70.8% [52.6–89], *P* = 0.03). The better survivals were due to the lower risk of relapse in this CloB2A2 AML subgroup (2‐year RI FB2A2: 41.2% [19–62.4] vs. CloB2A2: 16.7% [5–34.2], *P* = 0.05). This retrospective comparison suggests that the CloB2A2 RIC regimen can likely provide longer survival than that awarded by a FB2A2 RIC regimen and may become a new standard of care RIC regimen for allotransplanted AML patients. A prospective phase 3 randomized study is warranted.

## Introduction

In the myeloid setting, allogeneic stem cell transplantation (allo‐SCT) is indicated as consolidation for acute myeloid leukemia (AML) patients in first complete remission (CR1) with intermediate or high‐risk profile (defined by molecular genetic and cytogenetic alterations) 1, 2, or beyond CR1 1, 2 and for high‐risk myelodysplastic syndrome (MDS) patients (defined nowadays by the revised IPSS score) 3. While myeloablative conditioning regimen remains the standard of care for younger patients (<45 years), the development of reduced intensity conditioning (RIC) regimens 20 years ago has enabled transplantation of older AML/MDS patients or patients with comorbidities 4. Retrospective comparisons of both regimens have been associated with similar overall survival (OS) because of higher toxicity and higher nonrelapse mortality (NRM) for the former, and a higher relapse incidence (RI) for the latter 5, 6, 7. It took time for a better RIC regimen to be defined for such patients, and currently, the FB2A2 (fludarabine, 2 days of intermediate doses of busulfan and 2 days of antithymocyte globulin [ATG]) is considered as one of the standard RIC regimen in many centers worldwide, especially in France. Large series have shown OS between 37% and 76% and leukemia‐free survival (LFS) between 37% and 68% at 2–3 years posttransplant 8, 9, 10.

Recently, we have reported encouraging results of a clofarabine‐busulfan‐containing RIC regimen in adults with high‐risk AML/MDS in CR at the time of transplant where clofarabine replaced fludarabine as part of the FB2A2 regimen (CloB2A2), demonstrating a 2‐year OS and LFS of 75% and 69%, respectively 11.

Clofarabine acts by inhibiting ribonucleotide reductase and DNA polymerase, thereby depleting the amount of intracellular deoxynucleoside triphosphates available for DNA replication. Compared to fludarabine, clofarabine has an increased resistance to deamination and phosphorolysis, and hence better stability as well as higher affinity to deoxycytidine kinase (dCyd), the rate‐limiting step in nucleoside phosphorylation 12. Thus, CloB2A2 regimen may prove to be superior to the FB2A2 in patients with AML/MDS.

## Patients and Methods

### Study design and eligibility criteria

This was a multicenter retrospective study aiming to compare OS and LFS between AML/MDS patients receiving either CloB2A2 or FB2A2 RIC regimen for allo‐SCT, and reported to the SFGM‐TC registry between January 2009 and December 2014. No selection criteria other than those mentioned above were used for this study. Data were collected and investigators were requested to update the main outcomes, especially dates of relapse or death and of last follow‐up. The study was approved by the scientific committee of the SFGM‐TC, and performed according to their guidelines. During the study period, 355 patients from 26 French centers were identified, including 16 AML/MDS patients already reported as part of the previous prospective CLORIC study 11.

### Conditioning regimens

The FB2A2 consisted of 30 mg/m²/day fludarabine for 5 days (day‐6 to day‐2) combined with 3.2 mg/kg/day busulfan for 2 days (days‐3 and ‐2) and 2.5 mg/kg/day ATG (thymoglobulin) for 2 days (days‐2 and ‐1). In the other subgroup, the first 17 patients received a CloB2A2 regimen according to the previously published schedule 11 with 30 mg/m²/day clofarabine for 4 days (day‐8 to day‐5) combined with 3.2 mg/kg/day busulfan for 2 days (days‐3 and ‐2) and 2.5 mg/kg/day ATG for 2 days (days‐2 and ‐1). The 22 remaining patients received 30 mg/m²/day clofarabine for 5 days (day‐6 to day‐2) combined with 3.2 mg/kg/day busulfan for 2 days (days‐3 and ‐2) and 2.5 mg/kg/day ATG for 2 days (days‐2 and ‐1). Indeed, at the end of the prospective trial, the choice was made to add one more day of clofarabine with the hope to obtain even more antileukemic activity.

As GVHD prophylaxis, cyclosporine (CsA) alone was used in case of related donors in both groups, and for the 16 AML/MDS CloB2A2 patients treated within the previous prospective CLORIC trial 11, while CsA+ mycophenolate mofetil were used in case of unrelated donors.

### Statistical analyses

The primary endpoint of the trial was to compare 2‐year OS and LFS after allo‐SCT between the two groups (CloB2A2 vs. FB2A2). Secondary endpoints were relapse incidence (RI), nonrelapse mortality (NRM), acute and chronic GVHD rates, and comparison of 2‐year OS and LFS between both groups in the setting of AML or MDS patients. Clinical outcomes that were collected included demographic, disease and transplant characteristics, graft‐versus‐host disease (GVHD) status, time to relapse, and survival. FB2A2 patients were considered until December 2013 in order to allow sufficient follow‐up. Quantitative variables are described with median, range, and interquartiles range and were compared by a Wilcoxon rank‐sum test. Categorical variables are described with counts and percent and were compared by Wilcoxon test or Fisher exact test where appropriate. OS was defined as the time between the date of transplant and death. LFS was defined as survival without relapse. Probabilities of OS and LFS were calculated using the Kaplan–Meier estimate. Cumulative incidence functions (CIF) were used to estimate relapse incidence (RI) and nonrelapse mortality (NRM) in a competing risks setting, since death and relapse are competing together. In order to study acute and chronic graft‐versus‐host disease, we considered death and relapse as competing events. Acute and chronic GVHD were diagnosed and graded according to the standard criteria 13, 14. Survival probabilities are presented as percent and 95% confidence interval. Univariate analyses were done using log‐rank test for OS and LFS, Gray's test for CIF. Characteristics considered for univariate analysis were as follows: gender (male vs. female), type of disease (AML vs. MDS), type of RIC regimen (CloB2A2 vs. FB2A2), age at transplant (<vs. ≥60 years), year of transplant (< or ≥median), white blood count (WBC) at diagnosis (<vs. ≥5000/mm^3^), status at transplant (first complete remission [CR1] vs. others), type of donor (related vs. unrelated; female donor to male recipient vs. other situations), and cytomegalovirus (CMV) donor/recipient status (‐/‐ vs. others).

Multivariate analyses were performed using Cox proportional hazard model. Factors differing between two groups in terms of distribution and all factors significantly associated with one of the outcome studied were included in the multivariate analysis.

To allow for potential confounding factors between treatments that could influence outcome, propensity score matching was also performed, using the nearest neighbor matching. The following factors were included in the propensity score model: age, diagnosis (AML or MDS), status at transplantation (CR1 vs. others), donor type (HLA identical vs. unrelated donor), WBC at diagnosis (≥5000/mm^3^ vs. others), and CMV seronegativity in donor and recipient versus others. The purpose of the propensity score‐matching strategy was to reduce confounding effects of these variables, and strengthen causal inferences 15.

All tests were two‐sided and *P* ≤ 0.05 were considered as indicating significant association. Analyses were performed using the R statistical software version 3.2.3 (available online at http://www.R-project.org), and propensity score analysis was performed using the “MatchIt” package 16. Patients with missing values were excluded from the propensity analyses.

## Results

### Patients

During the study period, 316 AML/MDS patients were identified as having received a FB2A2 RIC regimen (56% males, median age: 59.2 years, AML 78.5%, CR1 72%, median follow‐up: 20 months) and 39 a CloB2A2 RIC regimen (62% males, median age: 60.8 years, AML 62%, CR1 69%, median follow‐up: 22.4 months) for allo‐SCT. The characteristics of patients (Table 1) were similar between groups except for the type of disease, the median year of transplant, and the CMV donor/recipient status. There were more MDS patients (38% vs. 21.5%, *P* = 0.01) in the CloB2A2 group, while there were more recipients with CMV‐positive status in the FB2A2 group (53% vs. 31%, *P* = 0.008). CloB2A2 patients have been transplanted more recently compared to other cases (median year of transplant: 2014 vs. 2012, *P* < 0.0001). A 2‐year OS, LFS, RI, and NRM for the whole cohort (*n* = 355) were 58% (52–64), 52% (47–58), 31% (26–36), and 16% (12–21), respectively.

**Table 1:** Characteristics of patients and donors in both groups

	CloB2A2 *N* = 39	FB2A2 *N* = 316	*P*‐value
Patients			
Gender: male	24 (62%)	178 (56%)	0.53
Median age at transplant: years (range)	60.8 (20.5–74)	59.2 (26–71.4)	0.84
Median follow‐up: months (range)	22.4 (10.5–67)	20 (1.18–61.8)	
Type of disease:			
Acute myeloid leukemia (AML)	24 (62%)	248 (78.5%)	
Myelodysplastic syndrome (MDS)	15 (38%)	68 (21.5%)	0.01
Cytogenetics for AML:			
Favorable risk	2 (9%)	12 (5%)	
Intermediate risk	16 (69%)	181 (79.5%)	
High risk	5 (22%)	35 (15.5%)	0.54
Missing	1	20	
White blood count at diagnosis: /mm^3^ (range)	3900 (*n* = 38) (700–165000)	5800 (*n* = 229) (100–2000000)	0.36
Status at transplant			
First complete remission	27 (69%)	229 (72%)	
Second or third complete remission	6 (15.5%)	56 (18%)	
Active disease	6 (15.5%)	31(10%)	0.67
Median interval between diagnosis and graft: months (range)	6.2 (3.5–155)	7.2 (1–243)	0.23
Median year of transplant (range)	2014 (2009–2014)	2012 (2009–2013)	<0.0001
Cytomegalovirus serology status: positive	12 (31%)	168 (53%)	0.008
Donors			
Gender: male	23 (59%)	201 (64%)	0.54
Female for male recipient	9 (23%)	57 (18%)	0.46
Sibling	17 (44%)	108 (34%)	0.25
Unrelated donor	22 (56%)	208 (66%)	
Cytomegalovirus serology status:			
Positive	15 (38.5%)	127 (40%)	0.83
Donor negative/recipient negative	20 (51%)	101 (32%)	0.01

#### Comparison of outcomes between both groups

The 2‐year OS was significantly higher in the CloB2A2 group (74.3% [60.5–88] vs. 55.8% [49.5–62.2], *P* = 0.03), but no differences between the two groups was observed in terms of 2‐year LFS (CloB2A2: 61.5% [46.3–76.8] vs. FB2A2: 51.1% [44.8–57.4], *P* = 0.20), 2‐year RI (28.2% [15.1–42.9] vs. 31.6% [26–37.3], *P* = 0.57), or 2‐year NRM (10.3% [3.2–22.2] vs. 17.3% [12.9–22.3], *P* = 0.24) (Table 2). Incidence of grade 2–4 or grade 3–4 acute GVHD were similar in both groups (FB2A2 23% vs. CloB2A2 18%, *P* = 0.39, and 8% vs. 3%, *P* = 0.25) as well as the 2‐year incidence of chronic GVHD (FB2A2 31.9% [26.2–37.6] vs. CloB2A2 26% [11.8–42.8], *P* = 0.42).

**Table 2:** Univariate analysis

Factors	2‐year OS	2‐year LFS	2‐year RI	2‐year NRM
Conditioning				
CloB2A2 versus FB2A2	74.3% (60.5–88) versus 55.8% (49.5–62.2) *P* = 0.03	61.5% (46.3–76.8) versus 51.1% (44.8–57.4) *P* = 0.20	28.2% (15.1–42.9) versus 31.6% (26–37.3) *P* = 0.57	10.3% (3.2–22.2) versus 17.3% (12.9–22.3) *P* = 0.24
AML versus MDS	59.4% (52.8–66) versus 53.3% (40.7–65.9) *P* = 0.59	55.4% (48.8–62.1) versus 42% (29.7–54.3) *P* = 0.04	27.5% (21.8–33.4) versus 43.8% (31.3–55.5) *P* = 0.01	17.1% (12.4–22.4) versus 14.2% (7.1–23.7) *P* = 0.57
Gender: Male versus female	56.6% (49–64.3) versus 59.8% (50.5–69) *P* = 0.39	51.1%(43.5–58.8) versus 54.2% (45.1–63.3) *P* = 0.49	30.6%(23.8–37.6) versus 31.7% (23.8–40) *P* = 0.82	18.3% (12.9–24.4) versus 14% (8.3–21.2) *P* = 0.19
Median age at transplant				
<60 years versus ≥60 years	62.5% (54.6–70.4) versus 53.4% (44.7–62.1) *P* = 0.17	56.2%(48.2–64.2) versus 48.4% (39.8–56.9) *P* = 0.24	28.6% (21.7–35.9) versus 33.8% (26.1–41.7) *P* = 0.47	15.2% (9.9–21.5) versus 17.8% (11.9–24.7) *P* = 0.53
Year of transplant				
<median versus ≥median	63.6% (55.1–72.1) versus 54.2% (40.8–67.5) *P* = 0.63	57.1% (48.3–65.8) versus 46.6% (33.9–59.4) *P* = 0.53	27.5% (19.9–35.6) versus 37.3% (25.6–49) *P* = 0.33	15.4% (9.7–22.4) versus 16.1%(10.2–23.1) *P* = 0.81
WBC^a^				
<5000/mm^3^ versus ≥5000/mm^3^	65.3% (56.2–74.4) versus 58.1% (48.8–67.4) *P* = 0.06	60.5% (51.4–69.7) versus 50.6% (41.2–59.9) *P* = 0.06	22.1% (15–30.1) versus 36.4% (27.6–45.3) *P* = 0.009	17.4% (11–25.1) versus 13% (7.5–20) *P* = 0.37
CR1 versus others status	61.7% (55–68.5) versus 47.5% (35.5–59.5) *P* = 0.02	56.3% (49.6–63) versus 41.3% (29.3–53.2) *P* = 0.08	31% (25–37.1) versus 32.4% (21.6–46.7) *P* = 0.71	12.7% (8.6–17.7) versus 26.3% (17–36.6) *P* = 0.003
Related versus unrelated donor	54.3% (44.5–64.2) versus 60.2% (52.9–67.5) *P* = 0.43	44.4% (34.8–54.1) versus 56.9% (49.6–64.1) *P* = 0.03	39.4% (30.1–48.4) versus 26.5% (20.4–33) *P* = 0.01	16.2% (9.7–24.1) versus 16.6% (11.6–22.4) *P* = 0.92
CMV donor/patient status: ‐/‐ versus others	61.7% (52.2–71.2) versus 56.1% (48.7–63.5) *P* = 0.64	54.1% (44.1–64.1) versus 51.6% (44.4–58.8) *P* = 0.62	35.8% (26.2–45.4) versus 28.7% (22.6–35.1) *P* = 0.42	10.1% (5.3–16.7) versus 19.7% (14.2–25.8) *P* = 0.06
Female donor to male recipient: Yes versus no	53.6% (39.3–68) versus 58.8% (52.4–65.3) *P* = 0.97	47.9% (34.3–61.6) versus 53.6% (47.1–60.1) *P* = 0.69	32.2% (20.1–44.8) versus 30.7% (25–36.6) *P* = 0.75	19.9% (10.4–31.7) versus 15.7% (11.4–20.8) *P* = 0.28

OS, overall survival; LFS, leukemia‐free survival; RI, relapse incidence; NRM, nonrelapse mortality; AML, acute myeloid leukemia; MDS, myelodysplastic syndrome; WBC, white blood count at diagnosis; CR1, first complete remission at transplant; CMV, cytomegalovirus.

a At diagnosis.

#### Univariate analysis

In univariate analysis, factors associated with significant higher OS were the CloB2A2 regimen (*P* = 0.03), and a CR1 status (*P* = 0.02) (Table 2). Factors associated with significant higher LFS were AML patients (*P* = 0.04) and use of an unrelated donor (*P* = 0.03). There were trends for better 2‐year OS and LFS in patients with less than 5000/mm3 WBC at diagnosis (*P* = 0.06 and *P* = 0.08, respectively). Significantly lower RI was observed in AML (vs. MDS) cases (*P* = 0.01) or in transplants from unrelated donors (*P* = 0.01).

#### Multivariate analysis

In multivariate analysis of the entire cohort, the FB2A2 RIC regimen was independently associated with lower OS (HR: 2.14, 95% CI: 1.05–4.35, *P* = 0.04) and a higher risk of relapse (HR: 2.17, 95% CI: 1.02–4.61, *P* = 0.04) (Table 3). There was a trend for lower LFS with the FB2A2 RIC regimen (HR: 1.75; 95% CI: 0.94–3.26, *P* = 0.08). CR1 status at transplant was associated with better OS (HR: 0.57, 95% CI: 0.36–0.91, *P* = 0.02), and LFS (HR: 0.61, 95% CI: 0.40–0.94, *P* = 0.03) while the use of an unrelated donor was associated with better LFS (HR: 0.68, 95% CI: 0.46–1.01, *P* = 0.05) and lower RI (HR: 0.53, 95% CI: 0.33–0.85, *P* = 0.008). Also, diagnosis of MDS was the other factor associated with higher RI (HR: 1.93, 95% CI: 1.07–3.47, *P* = 0.02). Finally, older age (≥60 years) was associated with significant lower OS (HR: 1.03, 95% CI: 1.00–1.05, *P* = 0.04).

**Table 3:** Multivariate analysis

Whole cohort	HR	95% CI	*P*‐value
OS			
FB2A2	2.14	1.05–4.35	**0.04**
MDS	0.97	0.54–1.73	0.91
CR1	0.57	0.36–0.91	**0.02**
Age ≥60 years	1.03	1.00–1.05	**0.04**
Unrelated donor	0.93	0.60–1.42	0.72
Donor/patient CMV‐/‐	1.12	0.73–1.71	0.61
WBC ≥5000/mm^3^ a	1	0.99–1.00	0.79
LFS			
FB2A2	1.75	0.94–3.26	0.08
MDS	1.45	0.87–2.40	0.15
CR1	0.61	0.40–0.94	**0.03**
Age ≥60 years	1.01	0.99–1.03	0.21
Unrelated donor	0.68	0.46–1.01	**0.05**
Donor/patient CMV‐/‐	1.07	0.72–1.61	0.73
WBC ≥5000/mm^3^ a	1	0.99–1.00	0.82
RI			
FB2A2	2.17	1.02–4.61	**0.04**
MDS	1.93	1.07–3.47	**0.02**
CR1	0.60	0.35–1.02	0.06
Age ≥60 years	1.00	0.98–1.03	0.75
Unrelated donor	0.53	0.33–0.85	**0.008**
Donor/patient CMV‐/‐	0.52	0.94–2.44	0.09
WBC ≥5000/mm^3^ a	1	0.99–1.00	0.60
NRM			
FB2A2	1.17	0.38–3.57	0.78
MDS	0.73	0.25–2.12	0.56
CR1	0.61	0.29–1.29	0.20
Age ≥60 years	1.04	0.99–1.08	0.10
Unrelated donor	1.16	0.55–2.44	0.69
Donor/patient CMV‐/‐	0.47	0.20–1.09	0.08
WBC ≥5000/mm^3^ a	1	0.99–1.00	0.82

OS, overall survival; LFS, leukemia‐free survival; RI, relapse incidence; NRM, nonrelapse mortality; MDS, myelodysplastic syndrome; WBC, white blood count at diagnosis; CR1, first complete remission at transplant; CMV, cytomegalovirus.

a At diagnosis.

#### Propensity score‐matching analysis

**Figure 1:**
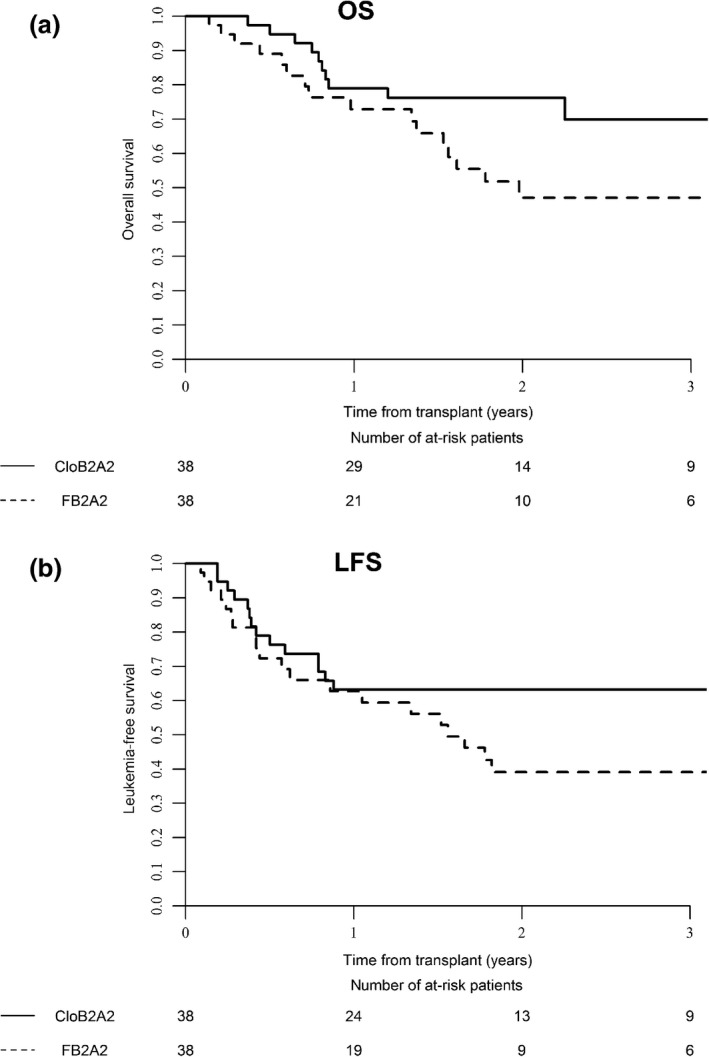
(A) Comparison of overall survival (OS) between patients receiving the CloB2A2 versus FB2A2 RIC regimen considering the whole cohort (acute myeloid leukemia [AML] + myelodysplastic syndrome [MDS] patients) and the propensity score‐matching strategy; (B) Comparison of LFS (leukemia‐free survival) between patients receiving the CloB2A2 versus FB2A2 RIC regimen considering the whole cohort (AML+MDS patients) and the propensity score‐matching strategy.

**Figure 2:**
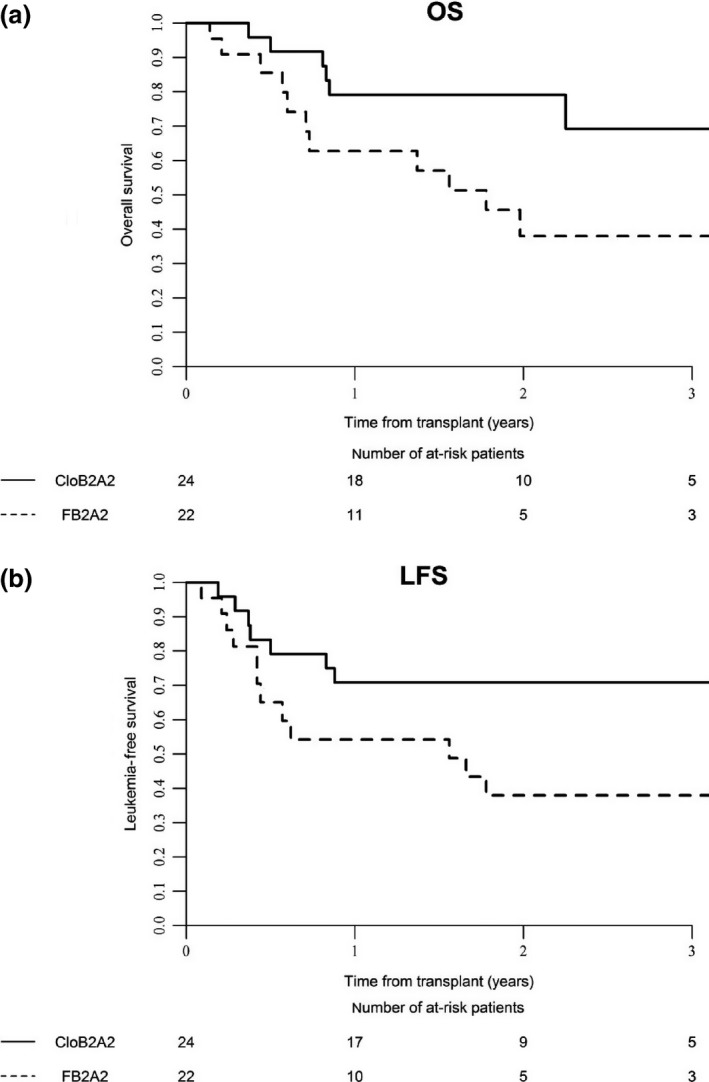
(A) Comparison of overall survival (OS) for acute myeloid leukemia (AML) patients receiving either the FB2A2 or the CloB2A2 RIC regimen considering the propensity score‐matching strategy; (B) Comparison of leukemia‐free survival (LFS) for AML patients receiving either the FB2A2 or the CloB2A2 RIC regimen considering the propensity score‐matching strategy.

In order to reduce confounding effects of variables, and strengthen causal inferences, a propensity score‐matching strategy was used to compare outcomes between the two groups (Table 4). Thirty‐eight patients were considered for comparison in each group (one patient with missing values in the CloB2A2 group was excluded from the analysis). CloB2A2 was associated with significant higher 2‐year OS (76.2% [62.7–89.8] vs. 47.1% [28.6–65.6], *P* = 0.02, Fig. 1A) and significant better LFS (63.2% [47.8–78.5] vs. 39.1% [21.9–56.3], *P* = 0.05, Fig. 1B). Considering only the AML subgroup, treatment with the CloB2A2 regimen remained associated with higher 2‐year OS (*P* = 0.01, Fig. 2A) and 2‐year LFS (*P* = 0.03, Fig. 2B) while lower RI (*P* = 0.05) was also observed with this regimen. In the MDS group, neither FB2A2 nor CloB2A2 RIC regimens were associated with OS, LFS, RI, and NRM.

**Table 4:** Comparison of outcomes between both groups when considering the propensity score‐matching strategy

Whole cohort	CloB2A2 *N* = 38	FB2A2 *N* = 38	*P*‐value
2‐year OS	76.2% (62.7–89.8)	47.1% (28.6–65.6)	0.02
2‐year LFS	63.2% (47.8–78.5)	39.1% (21.9–56.3)	0.05
2‐year RI	26.3% (13.5–41)	45% (27.1–61.3)	0.07
2‐year NRM	26.3% (13.5–41)	15.9% (5.6–31.1)	0.66

AML patients	CloB2A2 *N* = 24	FB2A2 *N* = 22	*P*‐value
2‐year OS	79.2% (62.9–95.4)	38% (14.5–61.6)	0.01
2‐year LFS	70.8% (52.6–89)	38% (16–59.9)	0.03
2‐year RI	16.7% (5–34.2)	41.2% (19–62.4)	0.05
2‐year NRM	12.5% (3–29.1)	20.8% (6–41.7)	0.58

MDS patients	CloB2A2 *N* = 14	FB2A2 *N* = 16	*P*‐value
2‐year OS	71.4% (47.8–95.1)	59.7% (32–87.4)	0.51
2‐year LFS	50% (23.8–76.2)	39% (11.3–66.7)	0.78
2‐year RI	42.9% (16.6–67)	52.9% (20.8–77.2)	0.70
2‐year NRM	7.1% (0.4–29.1)	7.1% (0.4–29.1)	0.98

AML, acute myeloid leukemia; MDS, myelodysplastic syndrome; OS, overall survival; LFS, leukemia‐free survival; RI, relapse incidence; NRM, nonrelapse mortality.

## Discussion

This large retrospective study is the first to compare a standard busulfan/fludarabine‐based RIC regimen (FB2A2) to a new RIC regimen where the fludarabine has been replaced by clofarabine (CLOB2A2) for allo‐SCT in AML/MDS patients. Although there are some limitations in our study (retrospective study, small number of patient in the CloB2 group, clofarabine patients were mostly enrolled on a small prospective trial), we showed here that the CLOB2A2 RIC regimen is well tolerated and provided significantly better survival for allografted AML patients, with a gain of more than 30% in terms of 2‐year OS and LFS compared to the use of the FB2A2 regimen. Such results seems to be explained by the better antileukemic activity of clofarabine, a second‐generation purine analog, which is known to have an increased resistance to deamination and phosphorolysis, and to induce a direct apoptosis of leukemic cells by activation of caspase 9 12, 17. Indeed, CloB2A2 RIC regimen allowed here to lower RI significantly as demonstrated by the multivariate analysis as well as the propensity score‐matching strategy. Also, a trend for lower NRM in the CLOB2A2 group could have participated to the better results observed with this regimen. Conversely, incidence of chronic GVHD did not explain such results, although the latter has been shown to have an important role in reducing relapse and improving LFS and OS 18.

OS and LFS were similar when comparing both regimens for MDS patients, a population that showed significantly higher incidence of relapse compared to AML cases. This highlights the fact that MDS and AML patients are two different entities with specific features 19, 20, and that clofarabine fails to overcome chemoresistance in the MDS setting. Higher busulfan dose intensity (FB4 reduced‐toxicity myeloablative regimen) may perhaps improve the outcome in MDS patients, as it has been demonstrated for AML cases in CR1/CR2 21, 22. However, one study retrospectively comparing FB2 versus FB3/FB4 regimens did not find a survival benefit for the latter in AML/MDS patients 9. Moreover, one has to keep in mind that the toxicity of myeloablative regimens is a contraindication to perform this type of regimen in older patients, as is the case for the majority of MDS/AML patients. Finally, a recent study evaluating the FB2 regimen in 114 CR1 older (median age 65 years) AML patients, but using 3 days of ATG (FB2A3), showed relatively poor outcomes, with a 2‐year OS and LFS of 48% and 42%, respectively, suggesting no advantage of higher dose of ATG as part of the FB2A2 RIC regimen 23. While myeloablative regimen should be considered currently as the standard of care for patients able to receive it 24, new strategies are definitely needed in the MDS setting 19.

It should be difficult to improve the results of CLOB2A2 treatment for AML patients (almost 80% of 2‐year OS and 71% of 2‐year LFS). For example, comparison overall of 4 versus 5 days of clofarabine was not associated with improved OS in this cohort (data not shown). Also, increasing clofarabine 25 or busulfan dose (CLOB3/B4) 26, 27 may be more toxic, while reducing the dose of ATG, if possibly associated with less relapse, may be more damaging in terms of severe acute or chronic GVHD 28. Strategies using clofarabine instead of high‐dose Ara‐C as part of the consolidation before transplant 29 or strategies to prevent relapse after transplant could be more appropriate 30.

Unfortunately, subgroup analyses according to molecular status or ELN classification for AML 1 could not be done here due to missing data.

In conclusion, the CloB2A2 RIC regimen can likely provide higher survival compared to the FB2A2 RIC regimen and may become the new standard of care RIC regimen for allotransplanted AML patient. A prospective phase 3 randomized study is urgently warranted.

## Conflicts of Interest

The authors declared no conflict of interest.
